# Noncovalent SUMO-interaction motifs in HIV integrase play important roles in SUMOylation, cofactor binding, and virus replication

**DOI:** 10.1186/s12985-019-1134-8

**Published:** 2019-04-02

**Authors:** Yingfeng Zheng, Kallesh Danappa Jayappa, Zhujun Ao, Xiangguo Qiu, Ruey-Chyi Su, Xiaojian Yao

**Affiliations:** 1 Laboratory of Molecular Human Retrovirology, University of Manitoba, Winnipeg, MB Canada; 2 Department of Medical Microbiology, Faculty of Medicine, University of Manitoba, Winnipeg, MB Canada

**Keywords:** Human immunodeficiency virus (HIV), Integrase (IN), SUMOylation, SUMO-interacting motifs (SIMs), LEDGF/p75, Protein-protein interaction, Nuclear localization, HIV replication

## Abstract

**Background:**

HIV integrase (IN) and its cellular cofactors, including lens-epithelium-derived growth factor (LEDGF/p75), Ku70, p300, and Rad52, are subject to small ubiquitin-like modifier (SUMO) modification. In addition to covalent SUMOylation, SUMO paralogs can also noncovalently bind proteins through SUMO-interacting motifs (SIMs). However, little is known about whether HIV IN contains SIMs and the roles of these motifs.

**Results:**

We searched for the amino acid sequence of HIV IN and investigated three putative SIMs of IN: SIM1 72VILV75, SIM2 200IVDI203 and SIM3 257IKVV260. Our mutational analysis showed that 200IVDI203 and 257IKVV260 are two bona fide SIMs that mediate IN-SUMO noncovalent interactions. Additionally, a cell-based SUMOylation assay revealed that IN SIMs negatively regulate the SUMOylation of IN, as well as the interaction between IN and SUMO E2 conjugation enzyme Ubc9. Conversely, IN SIMs are required for its interactions with LEDGF/p75 but not with Ku70. Furthermore, our study reveals that SIM2 and SIM3 are required for the nuclear localization of IN. Finally, we investigated the impact of IN SIM2 and SIM3 on HIV single cycle replication in CD4^+^ C8166 T cells, and the results showed that viruses carrying IN SIM mutants are replication defective at the steps of the early viral life cycle, including reverse transcription, nuclear import and integration.

**Conclusion:**

Our data suggested that the IN^SIM^-SUMO interaction constitutes a new regulatory mechanism of IN functions and might be important for HIV-1 replication.

## Background

HIV integrase (IN) is a key viral enzyme that catalyzes the integration of viral DNA into the host genome in all retroviruses. HIV-1 IN also functions in other key steps during the viral life cycle, including reverse transcription, nuclear import of the preintegration complex (PIC) and postintegration steps, such as viral protein expression, transcription, packaging and processing [1–4]. Similar to all retroviral INs, HIV-1 IN contains three canonical domains: an N-terminal HH-CC zinc-binding domain, a catalytic core domain (CCD) and a C-terminal DNA-binding domain, each with different individual functions. Full-length IN is a multimeric enzyme that functions as a tetramer [5]. IN undergoes multiple posttranslational modifications (PTMs) (e.g., ubiquitination, SUMOylation, acetylation and phosphorylation), which have been shown to play versatile roles in the functions of IN and HIV-1 viral replication [6–9]. The SUMOylation of IN has been published earlier [7]. However, much is still unknown about the physiological mechanisms of this modification.

Small ubiquitin-like modifier (SUMO) proteins are ~ 10 kD in size, and there are four subtypes (SUMO 1–4) in mammals, which are conserved among all eukaryotic cells [10]. SUMO 1, 2 and 3 are ubiquitous in cells, whereas SUMO4 is only expressed in certain tissues and organs [10, 11]. While SUMO2 and SUMO3 are 96% identical to each other, SUMO1 only shares 45% amino acid identity with SUMO2/3. SUMOylation is accomplished by a series of enzyme-catalyzed reactions [12]. Three enzymes are required for all SUMO modification pathways, including SUMO activating enzyme E1 (a heterodimer of Aos1 and Uba2), the unique E2 conjugating enzyme Ubc9 and a number of different E3 ligases, such as PIAS and RanBP2 [12]. In a previous study, Ubc9 was shown to interact with HIV-1 IN, and SUMO2/3 and Ubc9 negatively regulate the HIV-1 integration step [13]. Similar to ubiquitination, the substrate proteins can be poly-SUMOylated or mono-SUMOylated at single or multiple Lys targets. The outcomes of SUMO modification vary greatly from protein stability, cytosolic-nuclear translocation, and antagonizing other posttranslational modifications to transcriptional regulation [10].

During the SUMOylation, SUMOs are covalently conjugated to protein substrates through canonical four-amino-acid SUMO conjugation sites ψ-K-x-D/E (where ψ is a hydrophobic amino acid and x is any amino acid). HIV-1 IN was demonstrated to contain three SUMOylation sites (45LKGE, 135IKQE and 243WKQE) at three Lys residues (K46, K136 and K244), and the impairment of IN SUMOylation correlated with an early replication defect. Even though the mechanism underlying IN SUMOylation is not clear, it was hypothesized that SUMOylation might regulate the proximity between IN and its co-factors, which is indispensable for efficient viral replication [14]. IN has been shown to interact with multiple host proteins. Interestingly, a number of IN-interacting proteins, such as LEDGF/p75, Ku70, p300 and Rad52, are also SUMOylated [15–18]. LEDGF/p75 is one of the most important cofactors for IN [19]. Previous studies have shown that LEDGF/p75, as an IN-interacting protein, carried out multiple functions during HIV infection, including tethering IN to transcriptionally active regions of host chromosomes, enhancing the enzymatic activity of IN, stabilizing IN subunit-subunit interactions and promoting IN tetramerization and protecting IN from proteasomal degradation [20–33]. Interestingly, a previous study revealed that SUMOylation–defective IN mutants still retained LEDGF/p75 binding ability [7], and HIV IN SUMOylation mutations did not affect subcellular localization or viral DNA nuclear import [7]. Therefore, conclusive biochemical and functional data are still elusive in terms of the impacts of IN SUMOylation during HIV-1 viral replication.

SUMOs can also noncovalently interact with other proteins through specific SUMO-interacting motifs (SIMs). The most well characterized SIM is V/I-x-V/I-V/I or V/I-V/I-x-V/I/L, where x can be any amino acid in a parallel or anti-parallel orientation [34–36]. SUMO-modified proteins can interact with SIM-containing binding partners through noncovalent binding. Notable examples include the SIMs of human TRIM5α, which bind to SUMO-conjugated capsid protein and restrict M-MLV infection [37], and RanBP2 SIM mediates its binding with the complex of RanGAP1/SUMO1 and Ubc9 [38]. Overall, the functional consequences of the SUMO-SIM interaction vary considerably in different protein contexts, affecting protein SUMOylation, protein localization, and protein-protein interactions. (see a review [39]).

Interestingly, IN was shown to interact with SUMO1 and SUMO2 in a yeast two-hybrid system and a coimmunoprecipitation (co-IP) assay [13]. Thus, the intriguing questions to ask are whether IN bears SIMs and how the IN^SIM^-SUMO interaction modulates the multiprotein complex formation among IN and its SUMOylated cellular cofactors to affect different functions of IN. In this study, we examined the amino acid sequence of IN and defined two functional SIMs (200IVDI203 and 257IKVV260) in the catalytic core domain (CCD) and C-terminus. The SIMs of IN were shown to negatively regulate SUMOylation of IN, differentially modulate the IN-LEDGF/p75 and IN-Ku70 interactions, and contribute to the nuclear translocation step of HIV-1 IN. These findings not only uncover two possible SIMs of IN but also provide novel mechanistic insights into the regulation of multiple functions of IN by SIMs.

## Methods

### Cell lines and transfection

Human embryonic kidney 293 T and HeLa cell lines were cultured in Dulbecco’s Modified Eagle Medium (DMEM) supplemented with 10% fetal bovin serum (FBS) and 1% penicillin-streptomycin. Human CD4+ C8166 T-lymphoid cells were maintained in RPMI-1640 medium supplemented with 10% FBS and 1% penicillin-streptomycin. For the transfection of 293 T cells and HeLa cells, the standard calcium phosphate precipitation technique was used, as described previously [40] .

### Plasmids

To enhance the HIV IN expression, we have generated and used the codon-optimized IN pAcGFP-INopt plasmid in the study, which has been described elsewhere [8]. The constructs of GFP-INopt mutants M1 (V72A/I73A), M2 (I200A/V201A), M3 (V259A/V260A), M1 + M2 (V72A/I73A/I200A/V201A), M1 + M3 (V72A/I73A/V259A/V260A) and M2 + M3 (I200A/V201A/V259A/V260A) were synthesized through a two-step based PCR method using the GFP-INopt as a template and cloned into pAcGFP-C vector (Clontech) at BglII and EcoR1 sites [41]. The following primers were used for constructing various IN SIM mutants: 5′-IN-BglII primer: (5-TAAGATCTTCCTGGACGGCA-3) and 3′- IN-EcoR1 primer (5-GCTGAATTCTCAGTCCTCGTCCT-3); 5′-IN 72,3AA primer (5-AGGGAAAGGCTGCACTAGTGGCAGTG-3) and 3′-IN 72,3AA primer (5-CACTGCCACTAGTGCAGCCTTTCCCT-3); 5′-IN 200,1AA primer (5-AGGAGAGAGGGCAGCTGACATCATC-3) and 3′-IN 200,1AA (5-GATGATGTCAGCTGCCCTCTCTCCT-3); 5′-IN 259,60AA primer (5-AGCGACATCAAT GCAGCTCCTAGGCGGAAGG-3) and 3′-IN259,60AA primer (5-CCTTCCGCCTAGGAGCTGCATTGATGTCGCT-3). The IN 3KR mutant (K46R/K136R/K244R) and 3VI mutant (V72A/I73A, I200A/V201A, V259A/V260A) had been synthesized and cloned into the pUC57 vector by GenScript Inc. Then the IN 3KR or 3VI fragment was excised from pUC57-INopt with *BamH*I and cloned in frame at the 3′ end of the pAcGFP1-C vector with the same restriction enzyme. All the mutants were confirmed by sequencing.

SRa-HA-SUMO2 (plasmid 17,360) and pcDNA3/HA-SUMO3 (plasmid 17,361) were kindly provided by Dr. Edward Yeh from Addgene [41]. The pcDNA3-V5-Ubc9 was a generous gift from Dr. Ronald Hay (University of St. Andrews, St. Andrews, UK). The full-length wild-type Ubc9 was cut by *BamH*1/*Xho*I sites from pcDNA3-V5-Ubc9 and then cloned into CMV-HA vector. The constructed plasmid was named HA-Ubc9. SVCMVin-T7-LEDGF, and SVCMV-T7-Ku70 have been described previously [8, 19]. To construct pProLabel-Ku70, SVCMV-T7-Ku70 was digested with BamH1/Not1 and inserted into vector pProLabel which is derived from pProLabel-LEDGF [19].

HIV-1 HxBru RT/IN defective proviral plasmid (BruΔRI/R−/Gluc) used in this study was modified from a previously described Bru ΔRI/R- provirus [42]. To investigate the effect of IN SIM mutants on viral replication, mutant M2 (I200A/V201A) or M3 (V259A/V260A) was introduced into CMV-RT-IN express plasmid as described before [43].

### Antibodies and reagents

The rabbit anti-GFP polyclonal antibody (Molecular Probes Inc.) was used for immunoprecipitation. The antibodies for Western Blot (WB) were as follows: mouse anti-Ku70 (Abcam), horseradish peroxidase (HRP)-conjugated anti-GFP antibody (Molecular Probes), HRP-conjugated anti-HA antibody (Miltenyi Biotec.), and HRP-conjugated anti-T7 antibody (Novagen). The secondary antibody sheep anti-mouse IgG was purchased from Amersham Biosciences.

### Co-immunoprecipitation assay (co-IP)

The protocol for the co-immunoprecipitation assay studying the interaction between HIV-1 GFP-INwt/mut with HA-SUMO3/2 proteins was essentially as described [13], with minor modifications. Briefly, the cells after 40 h transfection were collected and washed with cold Phosphate buffered saline (PBS) once and then lysed in lysis buffer (20 mM Tris-HCl pH 7.5, 100 mM NaCl, 0.5% NP-40, 0.5 mM EDTA, 0.5 mM PMSF and protease inhibitor cocktail) on ice for 20 min. After centrifugation for 20 min at 14,000RPM to remove the cell debris, the cell lysates were precleared with protein G-Sepharose for 2 h, rotated with anti-HA mouse antibody at 4 °C for 3 h followed incubated with protein A sepharose overnight. The immunoprecipitates were washed with lysis buffer five times, and the bound proteins were separated in 12% SDS-PAGE gel and immunoblotted with anti-GFP antibody to detect HA-SUMO3/2-bound GFP-INwt/mut. 2% of the total celllysats were detected for the expressions of GFP-INwt/mut and HA-SUMO3/2.

The interactions between IN and various cellular proteins including T7-LEDGF/p75, T7-Ku70 or pProlabel-Ku70 and HA-Ubc9 were verified by co-IP as described above. Briefly, cell lysates from 293 T cells co-transfected GFP-IN and T7-LEDGF/p75, T7-Ku70, pProlabel-Ku70 or HA-Ubc9 were immunoprecipited with rabbit anti-GFP antibody. The IN-bound proteins were detected by a WB using anti-T7 or anti-HA antibody, or measured for ProLabel activity using the POLARstar OPTIMA multidetection microplate reader (BMG Labtech, Ortenberg, Germany) [8] (chemiluminescent co-IP system).Two percent of transfected cell lysates were used to detect the expressions of different proteins by WB using corresponding antibodies.

Detection of IN SUMOylation using immunoprecipitation analysis (SUMOylation assay) was described previously [44] with minor modifications. Two hundred and ninety-three T cells were cotransfected with HA-SUMO3 and GFP-IN wt/mut for 40 h. The cells were harvested and washed in cold PBS once and added 10 unit/ml Benzonase (Novagen, Billerica, MA, USA) in 2 mM MgCl_2_ for 20 min to reduce the cellular viscosity. Then the cells were lysed in 150 μl lysis buffer (0.15 M Tris-HCl, pH 6.7, 5% SDS, and 30% glycerol), which is then diluted 1:10 in PBS/0.5% NP40 plus complete protease inhibitor (Roche). Cell lysates were incubated with rabbit anti-GFP antibody for 2 h and followed by the protein A-Sepharose for another 2 h at 4 °C. The bound proteins were eluted with 4x Laemmli buffer and separated on SDS-PAGE gel. SUMO conjugated IN was detected by mouse anti-HA antibody in WB.

### Immunofluorescence assay

HeLa cells were grown on glass cover slips (12 mm^2^) in 24-well plates for 24 h and then transfected with different GFP-IN plasmids. After 48 h, cells on the cover slip were fixed and permeabilized for 30 min in methanol/acetone (1:1 ratio) at room temperature. The glass cover slips were incubated with a primary rabbit anti-GFP antibody followed by a secondary FITC-conjugated anti-rabbit antibody. Nuclei were stained with DAPI. Cells were visualized on a Carl Zeiss microscope (Axiovert 200) with a 63x oil immersion objective.

### Subcellular protein fractionation

Two hundred and ninety-three T cells were transfected with AcGFP-IN wt or 3VI in 6-well plate for 48 h. Cells were harvested and proteins were sequentially extracted, yielding cytoplasmic, nuclear and chromatin-bound fractions using a Thermo Scientific Subcellular Protein Fractionation Kit (Thermo Scientific, USA). Each fraction of proteins was subject to WB analysis using the anti-GFP antibody.

### Single cycle viral replication and infection

A HIV-1 single-cycle replicating virus was produced in 293 T cells as described previously [45]. Briefly, 293 T cells were co-transfected with an RT/IN /deleted HIV-1 provirus Bru∆RI/Gluc and each CMV-Vpr-RT-IN (wt or SIM M2 and M3 mutant) expression plasmid. After 48 h post-transfection, viruses were collected and concentrated from the supernatants by ultracentrifugation at 35,000 rpm for 2 h. Virus titers were quantified using HIV-1 p24 Antigen Capture Assay Kit (purchased from the NCI-Frederick AIDS Vaccine Program).

To test the effect of the IN mutants on viral infection, equal amounts of single cycle replicating viruses (adjusted by virion-associated p24 levels) were used to infect C8166 T cells 2 h, then washed and cells were cultured at 37 °C. At different time points after infection, the supernatant from each sample supernatants were collected and used for Gaussia luciferase Assay and HIV-1 p24 assay as described previously [45].

### Real-time quantitative PCR analysis

C8166 T cells were infected with above single cycle replicating virus harboring wild-type IN or IN SIM mutants. Heat-inactivated virus (pretreated at 65° for 30 min) was used as a negative control. 2 h after infection, the cells were washed twice and cultured in complete RPMI. At 12, 24 and 48 h post-infection, 1 × 10^6^ infected cells were harvested and subjected to DNA isolation using QIAmp blood DNA minikit (Qiagen). The total HIV-1 DNA, 2-LTR circle and integrated DNA levels were quantified in Mx3000P real-time PCR system (Stratagen, CA), with the protocols described previously [46].

## Results

### HIV-1 IN contains two SIMs required for efficient binding to SUMO3

**Fig. 1:**
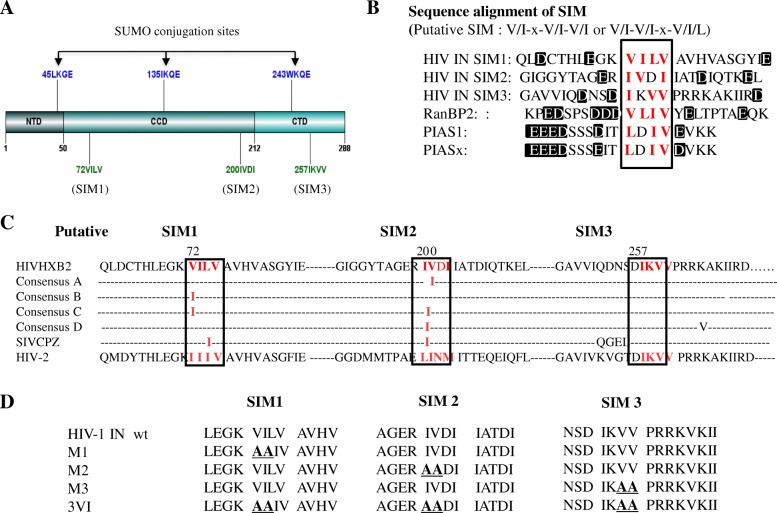
The three putative SIMs of IN and mutagenesis analysis. **a** Schematic of HIV-1 IN with its three putative SIMs and SUMO conjugation sites highlighted. IN contains three ψ-K-x-E SUMO conjugation sites (K46, K136 and K244) [7] and three putative SIMs. Four residues that constitute the hydrophobic core of each SIM are indicated (SIM1 72VILV75; SIM2 200IVDI203 and 257IKVV260). **b** The alignment of IN SIMs with other SIM-containing proteins, SUMO E3 protein RANBP2 and PIAS1 and PIASx. The SIM sequences are boxed with the hydrophobic amino acids highlighted in red. The acidic residues within 10 amino acids of the SIMs are shaded. **c** Three putative SIMs sequence alignment of different HIV-1 subtypes and HIV-2, SIVcpz integrase. **d** Mutagenesis analysis of IN SIMs. The point mutations introduced in the IN SIMs are shown in red. The IN SIMs were individually or simultaneously mutated to M1, M2, M3 or 3VI

Previous studies have shown that an SIM contains a hydrophobic core V/I-x-V/I-V/I or V/I-V/I-x-V/I/L and is often accompanied by negatively charged (acidic) residues, which enhance the SIM-SUMO interaction [34, 39]. We examined the amino acid sequence of HIV IN and found that it harbors three putative SIMs: SIM1 (72VILV75); SIM2 (200IVDI203) and SIM3 (257IKVV260) (Fig. 1a and b), and the sequence alignment of non-B HIV-1 subtypes, HIV-2 and SIVcpz, SIVmac were also shown that these putative SIMs are conserved (Fig. 1c). The sequence examination of these three putative IN SIMs and their surrounding amino acids revealed that all three sequences have acidic residues in close proximity to this motif (Fig. 1b). Several SIM-containing proteins, such as promyelocytic leukemia protein (PML), SUMO E3 ligase RANBP2 and PIAS family (PIAS1 and PIASx), share similar properties [47, 48] (Fig. 1b). To determine the importance of these motifs, we simultaneously or independently mutated these SIMs, resulting in the following mutants M1, M2, M3, 3VI, M1 + M2, M1 + M3 and M2 + M3 (Fig. 1d, data not shown for M1 + M2, M1 + M3 and M2 + M3). Each of these mutants was inserted into the GFP-IN plasmid [49]. Additionally, to differentiate IN SIMs from the SUMOylation sites of IN, we also generated the SUMOylation-defective IN mutant 3KR (K46R/K136R/K244R), which has three Lys residues within the SUMO conjugation sites (K46/K136/K244) of IN mutated to Arg residues (Fig. 1d) [7].

**Fig. 2:**
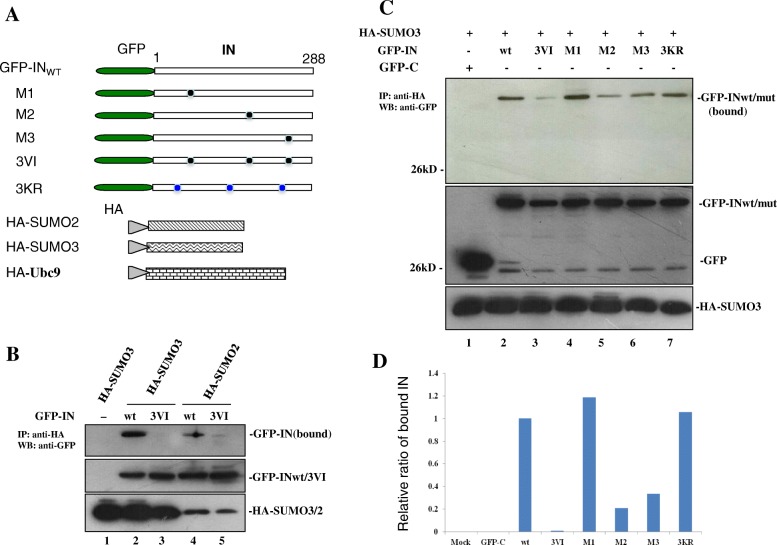
IN SIMs are required for the IN-SUMO2/3 interaction. **a** Schematics of various plasmid constructs encoding GFP-INwt/mut, HA-SUMO2, HA-SUMO3 and HA-ubc9. **b** 293 T cells were cotransfected with GFP-INwt/IN3VI and HA-SUMO2/3, the IN/SUMO interaction was analyzed by co-IP assay. Upper Panel: The HA-SUMO3/2 bound GFP-IN; Middle pane: The expression of GFP-INwt/3VI; Lower panel: The expression of HA-SUMO3/2 in 2% of total cell lysates. **c** SIM2 and SIM3 of IN mediate IN-SUMO3 interaction. Two hundred and ninety-three T cells were cotransfected with GFP-INwt/mut and HA-SUMO3 as indicated. Upper panel: The HA-SUMO3 bound GFP-INwt/mut; Middle and lower panels: The GFP-INwt/mut and HA-SUMO3 present in the 2% of the total cell lysates (middle panel on the left). **d** The relative ratio of pulled down GFP-INmut to GFP-INwt was calculated through densitometric analysis of the autoradiograms on **c**. The binding affinity of GFP-IN wt and HA-SUMO3 was arbitrarily set as 100%. The results are representative of two independent experiments

To verify whether these three SIMs in IN could indeed mediate IN/SUMO noncovalent binding, we first cotransfected GFP-IN wild type (wt) with either HA-SUMO2 or HA-SUMO3 (Fig. 2a) into 293 T cells to detect the IN/SUMO2 or IN/SUMO3 interaction. As negative controls, the cells were mock-transfected or transfected with HA-SUMO3 alone. By using a cell-based co-IP assay as described in Materials and Methods, we found that GFP-INwt had strong binding affinity with HA-SUMO3 and HA-SUMO2 (Fig. 2a, lane 2 and lane 4), whereas the GFP-IN 3VI mutant, which has six hydrophobic amino acids mutated within all three SIMs, did not bind either HA-SUMO3 or HA-SUMO2 (Fig. 2a, lane 3 and lane 5). To further pinpoint which SIM(s) of IN actually mediate the noncovalent binding of IN/SUMO3, 293 T cells were cotransfected with HA-SUMO3 and GFP-INwt or different IN SIM mutants 3VI, M1, M2 and M3 (Fig. 2b). In parallel, the cotransfection of HA-SUMO3 with GFP-C or GFP-IN 3KR was used as a control. The interactions between HIV-1 GFP-INwt or different IN mutants with HA-SUMO3 were analyzed by co-IP assay. The results revealed that the GFP-IN mutants M1 and 3KR retained similar IN/SUMO3 binding ability with GFP-INwt, whereas the IN SIMs mutants, including 3VI, M2 and M3, were impaired for IN-SUMO3 binding to different extents (Fig. 2b, compare lane 2 with lane 3, 5, and 6). The densitometric analysis (data not shown) suggest M2 and M3 mutants have 80 and 67% reduction in IN/HA-SUMO3 binding affinity, respectively. Taken together, these data demonstrated that HIV-1 IN is capable of interacting directly with SUMO2 and SUMO3, and at least two SIMs in IN (SIM2 and SIM3) are involved in the noncovalent binding of IN/SUMO3.

### HIV IN SIM mutants decrease its own SUMOylation and Ubc9 binding

**Fig. 3:**
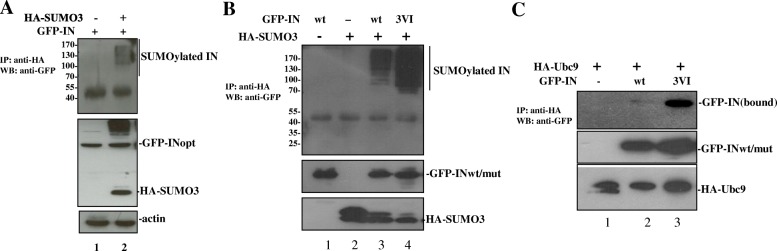
HIV IN SIM mutants decrease its own SUMOylation and Ubc9 binding. **a** IN is subject to SUMO3 modification. The 293 T cells were cotransfected with GFP-INwt and HA-SUMO3 or HA-SUMO3 alone. The SUMOylation of IN by HA-SUMO3 was detected by the SUMOylation assay as described in the Material and Methods. The SUMOylation level of IN is shown in the upper panel. Additionally, 2% of the total protein exacts were loaded onto SDS-PAGE gels to detect GFP-INwt and HA-SUMO3 expression (middle panel). Endogenous beta-actin was detected as a loading control (lower panel). **b** The IN 3VI mutant had higher SUMOylation levels than INwt. The 293 T cells were cotransfected with different plasmids as indicated, and the SUMOylation of GFP-INwt and 3VI was analyzed by using a SUMOylation assay. The SUMOylation level of INwt/mut is shown in the upper panel. The expression levels of GFP-INwt/mut and HA-SUMO3 are shown in the middle and the lower panels, respectively. **c** The interaction between IN and SUMO E2 protein Ubc9 was increased in IN SIMs mutant 3VI. HA-Ubc9 was cotansfected with GFP-INwt/3VI plasmids in 293 T cells and subjected to co-IP assay to study the binding affinity of IN-Ubc9. GFP-INwt/3VI bound HA-Ubc9 was shown in the upper panel. In addition, 2% of total cells were lysed to check the expression of GFP-INwt/3VI and HA-Ubc9 (middle and lower panels)

Substrate protein interactions with SUMO through SIM(s) have been shown to regulate the SUMOylation of different proteins (see review [39]). To study the potential effect of IN/SUMO binding on its own SUMOylation, the SUMOylation of IN was first confirmed by a cell-based SUMOylation assay as described in materials and methods. The results showed that multiple shifted bands of SUMOylated IN were detected in cells transfected with HA-SUMO3 and GFP-IN (Fig. 3a, lane 2, upper panel). The similar intracellular expression levels of GFP-INwt were verified by WB with the corresponding antibodies (Fig. 3a middle panel). To further test whether IN^SIM^-SUMO binding regulates its own SUMOylation, the SUMOylation levels of GFP-INwt and 3VI, were examined by the SUMOylation assay (Fig. 3b). As negative controls, cells transfected with GFP-INwt or HA-SUMO3 alone were included. Unexpectedly, the results showed that the SUMOylation level of the SIM mutant 3VI was approximately four times greater than that of GFP-INwt (Fig. 3b, compare lane 4 to lane 3), suggesting that IN^SIM^-SUMO binding negatively regulate the SUMOylation of IN.

Since the SUMO E2-conjugating enzyme Ubc9 is indispensable for the SUMOylation of all proteins, and HIV-1 IN was shown to interact with Ubc9 in a previous study [13], we next tested whether IN/SUMO binding via SIMs has any effect on the IN/Ubc9 interaction, thus negatively regulating the SUMO conjugation of IN. We assessed the interaction between HA-Ubc9 and the GFP-INwt or 3VI mutant using cell-based co-IP assays [8]. The data revealed that the GFP-IN3VI mutant had greatly increased IN/Ubc9 binding when compared with GFP-INwt (Fig. 3c, upper panel, compare lane 3 with lane 2). The increased binding ability of GFP-IN3VI with HA-Ubc9 is consistent with the increased SUMOylation level of 3VI (Fig. 3b, lane 4). Above observation suggest that IN-SUMO noncovalent binding through its SIMs disfavors its own SUMOylation.

### IN SIMs differentially regulate IN-LEDGF/p75 and IN/Ku70 interactions

**Fig. 4:**
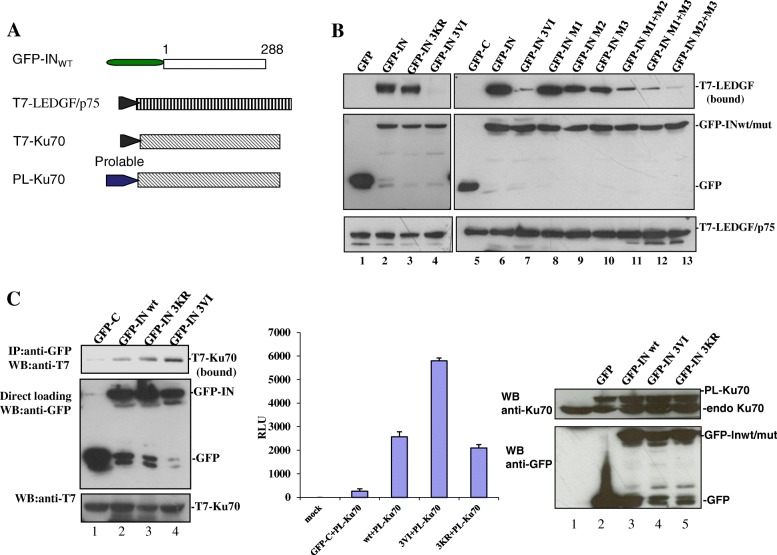
IN SIMs differentially regulate their interaction with LEDGF/p75 and Ku70. **a** Schematics of various plasmid constructs encoding GFP-INwt, T7-LEDGF/p75, T7-Ku70 and ProLabel-Ku70 (PL-Ku70). **b** The interaction of T7-LEDGF/p75 with GFP-INwt or mutants (3KR, 3VI, M1, M2, M3, M1 + M2, M1 + M3 and M2 + M3) was analyzed by co-IP assay. The GFP-IN-bound T7-LEDGF/p75 was detected by immunoprecipitation of the cell lysates with an anti-GFP antibody and immunoblotting with an anti-T7-HRP antibody (upper panel). The expression of GFP, GFP-INwt/mut and T7-LEDGF/p75 are shown in the middle and lower panels. The results are representative of three independent experiments. **c** The interaction of Ku70 with INwt/mutant was detected by co-IP assay. Left panel: The upper panel showed the bound T7-Ku70 in each sample. Two percent of cell lysates were used to detect the expression of GFP, GFP-INwt/mut and T7-Ku70 by WB (middle panel and lower panel). Middle and right Panels: The interaction of ProLabel-Ku70 (PL-Ku70) with GFP-INwt/mut was detected by chemiluminescent co-IP assay. The chemiluminescent signals from IN-bound PL-Ku70 present in the complexes were measured using the ProLabel Detection Kit II and valued as relative luminescence units (RLU). The results are representative of two independent experiments. Expression levels of PL-Ku70 and GFP-IN wt/mut or GFP alone in each sample were analyzed by anti-Ku70 and anti-GFP-HRP antibodies (right panel)

The SIMs present in the protein mediate protein-protein interactions through the binding between SIM-containing proteins and their SUMOylated partners. For example, the SIM of RANBP2/Nup358 was shown to mediate the interaction between RANBP2/Nup358 and SUMOylated RanGAP1 [35]. To investigate whether SIMs in HIV IN could play a role in IN interacting with its SUMOylated cofactors, we investigated the effect of different IN SIM mutants on their binding to LEDGF/p75 and Ku70, as both LEDGF/p75 and Ku70 are SUMOylated proteins [15, 16]. To this end, we first cotransfected 293 T cells with T7-tagged (T7-)LEDGF/p75 (Fig. 4a) [19] and GFP-INwt or the two IN mutants 3VI and 3KR and examined their interactions using the above described co-IP assay. The results showed that the IN SUMOylation defective mutant 3KR bound T7-LEDGF/p75 to the same extent as INwt (Fig. 4b, compare lanes 3 and 2), which is consistent with the previously reported data [7]. However, IN SIM mutant 3VI completely lost the ability to bind with T7-LEDGF/p75 (Fig. 4b, lane 4). To investigate which SIM(s) of IN is required for IN-LEDGF/p75 binding, different IN SIM mutants, including M1, M2, M3 and M1 + M2, M1 + M3, and M2 + M3, were individually cotransfected with T7-LEDGF/p75 into 293 T cells and processed with a co-IP assay. The results showed that the IN M1 mutant retained the ability to bind LEDGF/p75 with a similar affinity as INwt, whereas IN-LEDGF/p75 binding affinities were compromised to different extents in other IN SIM mutants (3VI, M2, M3, M1 + M2, M1 + M3, and M2 + M3) (Fig. 4b, lanes 9–13). Among these IN SIM mutants, M2 and M3 have 40–60% binding affinity compared to INwt, whereas M1 + M2 and M1 + M3 mutants only showed approximately 10–20% of the binding level of INwt; 3VI and M2 + M3 mutants displayed less than 10% of the binding affinity level of INwt-LEDGF. These data suggest that both IN SIM2 and SIM3 mutants compromise IN-LEDGF/p75 interaction.

Ku70 is another IN-interacting protein that is subject to SUMO modification [15], we next analyzed the effect of SIM in IN on the IN-Ku70 interaction. Briefly, a plasmid expressing GFP-INwt, 3KR, or 3VI was cotransfected with T7-Ku70 (Fig. 4a) [8] in 293 T cells. The interaction of IN-Ku70 was first verified by the co-IP assay. The results showed that the IN mutant 3KR and INwt displayed similar binding to T7-Ku70, whereas the IN mutant 3VI had two-fold increased binding affinity for Ku70 (Fig. 4c left panel). Additionally, above observation was confirmed by using chemiluminescent co-IP system, as described previously [19]. Briefly, GFP-INwt, 3KR, or 3VI was cotransfected with ProLabel-Ku70 in 293 T cells. The interaction of IN-Ku70 was verified by chemiluminescent co-IP assay as described in the Materials and Methods. Consistent with the above observation, the experiment confirmed that IN mutant 3KR and INwt bind PL-Ku70 with similar binding affinities, whereas the 3VI mutant had an increased binding affinity for Ku70 (Fig. 4c middle panel), even though similar expression levels of GFP-INwt and mutants 3KR and 3VI were detected in the cells (Fig. 4c right panel). These results indicate that IN SIMs differentially regulate IN-LEDGF/p75 and IN-Ku70 binding.

### IN SIMs influence the intracellular localization of the IN protein

**Fig. 5:**
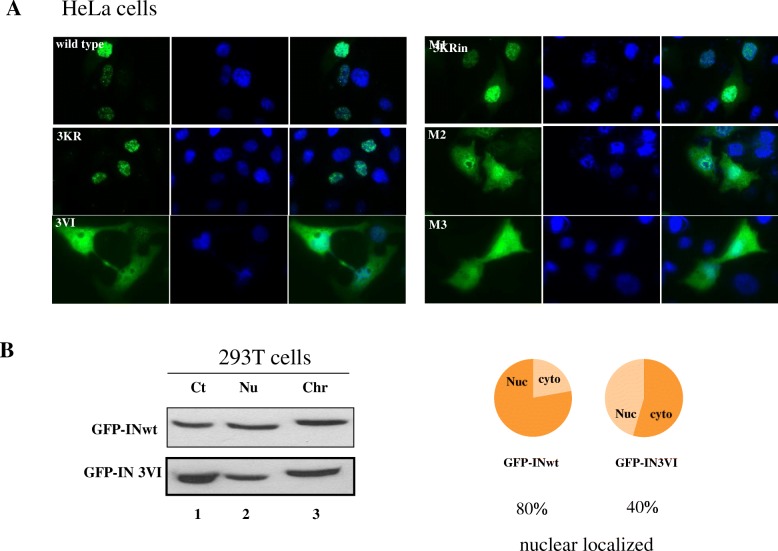
HIV IN SIMs affect the nuclear localization of IN. The potential effects of IN SIMs on the nuclear localization of IN were evaluated in HeLa cells. **a** HeLa cells transfected with different GFP-INwt/mut (green) were analyzed for their subcellular localization by indirect fluorescence assay. The nuclei were stained with DAPI (blue). **b** 293 T cells were transfected with GFP-INwt or 3VI for 48 h. The subcellular distribution of INwt and 3VI was determined by the subcellular fractionation method. Ct, cytoplasmic extracts; Nu, nuclear extracts; Chr, chromatin bound extract. The ratio of nuclear portion (a combination of Nu and Chr from left panel) to the cytoplasmic fractionate of proteins was calculated and shown as a pie chart in the right panel. The nuclear localized portions of GFP-INwt and 3VI in the total protein extracts are approximately 80 and 40%, respectively

Previous studies have revealed that the SUMO-SIM interaction affects the subcellular localization of SIM-containing proteins [50, 51], and the SUMOylation of IN plays a role in the subcellular localization of IN [13]. To study whether IN SIMs affect the localization of IN inside cells, HeLa cells were transfected with different IN plasmids, including GFP-INwt, 3KR, 3VI, M1, M2 and M3 and the localization of IN protein was observed by Immunofluorescence assay. The results showed that GFP-INwt and the 3KR and M1 mutants were exclusively localized to the nucleus, while the mutants 3VI, M2 and M3 showed both cytoplasmic and nuclear localization (Fig. 5). The nuclear localization pattern of IN 3KR is in line with a previous report showing that the SUMO modification of IN at three Lys residues (K46/K136/K244) is not required for its nuclear translocation [7]. However, the distinct localization pattern of the IN SIM mutants 3VI, M2 and M3 suggested the involvement of SIM2 and SIM3 in the nuclear translocation of IN proteins. To further confirm the impaired nuclear localization of IN mutant 3VI, we further used protein fractionation method to separate GFP-INwt or 3VI transfected 293 T cells into cytoplasmic, nuclear and chromatin-bound portions. The presence of GFP-INwt and 3VI in these particular cellular compartments was detected by WB analysis (Fig. 5b, left panel). The ratios of the levels of INwt and 3VI present in the nuclear portion (a combination of nuclear and chromatin-bound from the left panel) to the cytoplasmic fractionate were calculated from their relative protein band intensities and shown as a pie chart (Fig. 5b, right panel). The nuclear portion of GFP-INwt and 3VI in the total protein extracts was approximately 80 and 40%, respectively. Taken together, the results suggested that the SIM2 and SIM3 are required for efficient IN nuclear translocation.

### HIV-1 bearing IN SIM mutations is defective at the early steps of replication

**Fig. 6:**
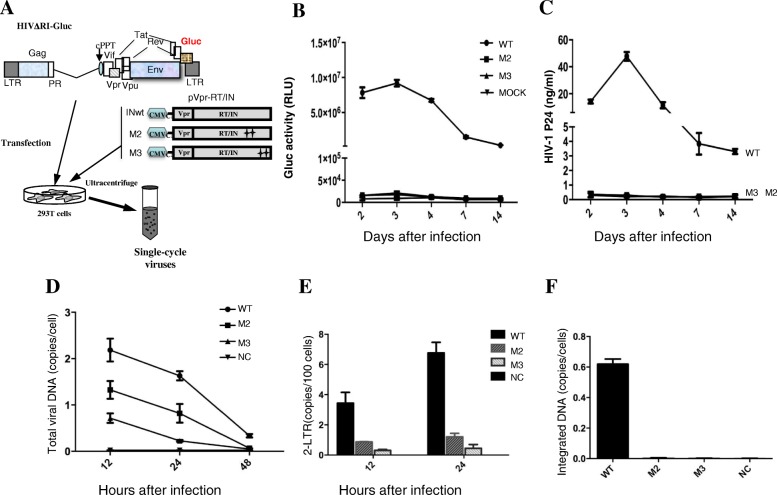
HIV-1 carrying IN SIM mutants are replication defective at the early stages of viral replication, including reverse transcription, nuclear import and integration. **a** Schematic structure of HIV-1 RT and IN deletion provirus HIVΔRI/Gluc and the CMV-Gag-Pol-expressing plasmid harboring INwt, mutant M2, or M3. Single-cycle replicating viruses harboring INwt or mutants M2 and M3 were produced from cotransfected 293 T cells, collected by ultracentrifugation and normalized by virion-associated p24 levels. Then, equal amounts of each virus stock were used to infect C8166 T cells. At various time intervals, HIV-1 replication was determined by Gaussia luciferase assay (**b**) and HIV-1 p24 ELISA (**c**). To determine the step of viral replication that was affected, real-time quantitative PCR analysis was performed to detect the total HIV-1 DNA (**d**), 2-LTR circle (**e**) and integrated DNA levels (**f**) at various times post infection, as indicated

Our above results suggested that HIV-1 IN has two SIMs (SIM2 200IVDI203 and SIM3 257IKVV260) that affect the nuclear localization of IN and are required for LEDGF/p75 binding. We next wanted to examine the role of IN SIMs during viral replication. Briefly, we introduced SIM2 or SIM3 mutations into a previously described HIV single-cycle infection system [42] and evaluated viral replication. Briefly, IN M2 or M3 mutations were introduced into a plasmid expressing the Vpr-RT-IN fusion protein and cotransfected with an RT/IN-deleted HxBruR−/RI/E+/Gluc+ plasmid [45]. In this provirus, the HIV-1 *Nef* gene was replaced by a gene encoding secreted Gaussia luciferase (GLuc) (Fig. 6a). In parallel, a wild type IN (WT)-encoded single-cycle virus was also produced. To test the infectivity of IN SIM mutant viruses, CD4+ T C8166 cells were infected with an equal amount of each IN wt/mut virus (normalized by P24^gag^). At different time points, HIV-1 replication was monitored by measuring Gluc activity (Fig. 6b) and the level of HIVp24^gag^ (Fig. 6c) in the supernatants. As shown in Fig. 6b and c, in contrast to the wild type virus, there were very low levels of the produced Gluc activity, and HIVp24 could be detected in cell cultures infected with viruses (M2 and M3) harboring IN mutants, M2 or M3. These results indicate that IN SIM mutant virus M2 or M3 is replication defective.

To further define which step(s) of the HIV-1 replication cycle were affected, we analyzed viral reverse transcription, nuclear import and integration steps in C8166 T cells infected with IN SIM mutant viruses by real-time PCR. The results showed that the viral cDNA synthesis in HIV-1 M2 and M3 mutant virus-infected cells were decreased to 2- and 3-fold, respectively, compared to that of wild-type virus infected cells (Fig. 6d). Moreover, the levels of 2-LTR circles in M2 and M3 mutant virus-infected C8166 cells were approximately 4- and 11-fold lower, respectively, than that of the wild-type virus (Fig. 6e). As expected, the integrated proviral DNA could not be detected in either M2 or M3 virus-infected samples (Fig. 6f), which is well correlated with the replication defects of M2 or M3 mutant virus. All of these data suggest that the SIMs present in HIV IN are required for the early establishment of viral infection, including reverse transcription, nuclear import, and integration.

## Discussion

In this study, we report that HIV-1 IN bears two functional SIMs (SIM2 200IVDI203 and SIM3 257IKVV260), that negatively regulate the SUMOylation of IN, as well as the interaction between IN and SUMO E2 conjugation enzyme Ubc9. Also. The results indicate that SIMs in IN are required for its interaction with LEDGF/p75, but not with Ku70. Viruses carrying IN SIM mutants showed impaired viral reverse transcription, nuclear import and integration steps, resulting in defective replication. This report provides the first evidence for the roles of noncovalent SIMs in HIV IN for its functions during viral replication.

Sequence analysis revealed that the three putative SIMs (SIM1 72VILV75, SIM2 200IVDI203 and SIM3 257IKVV260) present in IN conform to this consensus (Fig. 1b). However, our mutational analysis suggests that SIM2 and SIM3 present the major sites for the IN-SUMO interaction. Within these two IN sequences, the hydrophobic residue I200 is implicated in both chromatin and LEDGF/p75 binding [19]; V260 is involved in the multimerization and structural stabilization of IN [52, 53]. In this study, the SIM2 mutant I200A/V201A had decreased IN-LEDGF/p75 binding affinity (Fig. 4b), which is consistent with a previous report [19]. However, it remains an open question as to whether the IN^SIM^-SUMO interaction has any impact on its chromatin association or multimerization. Remarkably, V201I, the mutant sequence that still conforms to the consensus SIM, occurs as a natural polymorphism in drug-naïve patients [54, 55]. This conservative substitution within SIM2 200IVDI203 thus highlights the importance of this sequence in the functions of IN and HIV-1 replication.

Noncovalent SUMO binding or the SIM-SUMO interaction has been shown to facilitate SUMOylation of SIM-containing proteins [50, 56–58]. For example, the interaction between the SIM of Sp100 and SUMO-Ubc9 enhanced the SUMOylation of Sp100 [56]. This SIM-dependent SUMOylation has also been described in various SUMO targets, such as Daxx, RANBP2/Nup358, HIPK2 and BLM [50, 57, 58]. Interestingly, our results also showed that the IN SIM mutant 3VI has increased SUMOylation levels compared with INwt, suggesting that IN SIMs negatively regulate SUMOylation (Fig. 3b). Another line of evidence from this study also strongly supports this conclusion. The mutant 3VI had significantly increased Ubc9 binding ability compared with INwt (Fig. 3c). Ubc9 directly binds the SUMO conjugation consensus ψ-K-x-D/E sequence in substrates [59, 60], and mutating ψ-K-x-D/E abolishes both Ubc9 binding and the SUMOylation of substrate proteins [60]. Similarly, a previous study reported that Srs2 SUMOylation inhibits its noncovalent SUMO binding, possibly due to the reduced availability of its SIM motif for interactions with SUMOylated proteins in general by Srs2 SUMOylation [61]. Thus, it appears that noncovalent SUMO binding and covalent SUMO modification or SUMOylation can mutually regulate with each other, either positively or negatively.

The functional outcomes for noncovalent SIM/SUMO binding vary and are largely dependent on SUMOylated proteins and SIM-containing binding partners. SUMO binding through SIM(s) affects protein stability, cytosolic-nuclear translocation, and transcriptional regulation through altered protein-protein or protein-DNA interactions at the molecular level (see a review [10]). To investigate whether the IN^SIM^-SUMO interaction could mediate the binding of IN with its SUMOylated cellular cofactors, we tested the interactions between HIV-1 IN with two SUMOylated proteins, LEDGF/p75 and Ku70. Our data found that while the IN SIM mutant 3VI was severely impaired in IN-LEDGF/p75 binding, it showed a two-fold increase in IN-Ku70 binding affinity (Fig. 4b and c). Additionally, 3VI bound Nup62, a non-SUMOylated cofactor of IN [62], at the same level as INwt (data not shown). These results together imply that IN SIMs might be involved in the regulation of the interaction between IN and SUMO-conjugated binding partners. Closer examination revealed that the SIM1 mutant M1, which still binds SUMO3, retains the full ability to interact with LEDGF/p75 (Fig. 4b). This observation thus strengthens the notion that SIM2 and SIM3 of IN are required for the IN-LEDGF/p75 interaction. However, we cannot formally exclude the possibility that creating six point mutations in 3VI might have profound impacts on other functions of IN, which may indirectly influence the IN-LEDGF/p75 or IN-Ku70 interaction. For example, V260 has been shown to be critical for the multimerization of IN [53], and I200 is required for the chromatin binding ability of IN [63]. The SIM-SUMO interaction has been shown to influence the subcellular localization of SIM-containing proteins. A prominent example is the sequestration of Daxx to PML nuclear bodies, which is mediated through the binding of the SIM located at the C-terminus of Daxx and SUMOylated PML [50]. Another example is that the SIMs of PML, Sp100 and hDaxx are required for the recruitment of these proteins to herpes simplex virus type 1 (HSV-1)-induced foci, which also recruit SUMO proteins and SUMO E3 ligase PIAS2β [51]. In the present study, our results from HeLa cells and 293 T cells revealed that the IN SIM mutant 3VI was severely impaired in its nuclear localization (Fig. 5a and b). More specifically, SIM2 200IVDI203 and SIM3 257IKII260 but not SIM1 72VILV75 are involved in the nuclear import of IN proteins (Fig. 5a). As both SIM2 and SIM3 mutants that do not exclusively localize to the nuclei are also impaired for IN-SUMO3 and IN-LEDGF/p75 interactions, and LEDGF/p75 was initially reported to be indispensable for the nuclear import and chromosomal targeting of IN [31, 33], the cytoplasmic localization pattern of SIM2 and SIM3 mutants might be the reason or result of impaired IN-LEDGF/p75 interaction and/or chromatin association. In accordance with this hypothesis, SIM2 mutant I200A was shown to be defective for chromatin binding in a previous report [19]. Second, we also consider the possibility that mutations introduced to IN SIMs might block the recruitment of SUMOylated cellular cofactor(s) of IN, which is required for the nuclear translocation of IN proteins. RANBP2 (Nup358) is one of the candidates that is SUMOylated and might be involved in the nuclear import of IN. RANBP2 is a nucleoporin with SUMO E3 ligase activity that harbors both SIMs and SUMO conjugation sites [35, 57, 63, 64]. As an SUMO E3 ligase, this protein has been shown to promote the SUMOylation of a number of SUMO targets, including Mdm2, HDAC4, topoisomerase II-alpha, PML, or Sp100 [57, 65–67]. Additionally, RANBP2 has been shown to be critical for HIV-1 replication and is involved in the nuclear import of the PIC and integration, both of which are closely linked with the functions of the HIV-1 IN [68, 69]. We may speculate that SUMOylated proteins, such as RANBP2, might form a complex with IN through binding SIMs and function in the nuclear import of IN. This possibility requires further experimental investigation.

It is known that introducing numbers of IN mutations in the context of virus may cause pleiotropic effects during HIV-1 replication [1, 70]. In order to limit such pleiotropic effects, we have used a previously described single cycle replicating virus system, in which the viral reverse transcriptase and integrase were complemented *in trans* [43] (Fig. 6a). Through the analyses with this HIV single cycle replication system, it showed that two functional SIMs (SIM2 200IVDI203 and SIM3 257IKVV260) are required for the multiple steps, including reverse transcription, nuclear import, and integration, in the early stage of HIV infection (Fig. 6). These observations are well correlated with the fact of SIMs are important in regulation of its SUMOylation, interacting with LEDGF/p75, and its ability for the nuclear import. Indeed several previous studies shown that the disruption of HIV integrase binding to endogenous LEDGF can significantly interfere with the progeny virus infectiousness [71, 72]. Another study also reported that a compound, LEDGINs, which specifically inhibit IN interaction with LEDGF/p75, was able to disrupt virus assembly and lead to the large portion of progeny virions display aberrant morphogenesis [73]. Whether the SIMs in the IN play similar roles during HIV morphogenesis still wait for more detailed studies.

## Conclusions

In conclusion, our data show that IN SIMs (200IVDI203 and 257IKVV260) bind to SUMO-2 and SUMO-3, facilitating IN^SIM^-SUMO interactions, and are involved in the regulation of their own SUMOylation, cofactor binding, and multiple steps during the early stage of HIV replication. Further studies are needed to elucidate the molecular basis of IN SIMs in the regulation of these multiple functions of IN.

## Abbreviations

CCD: Catalytic core domain
co-IP: coimmunoprecipitation
GLuc: Gaussia luciferase
IN: Integrase
LEDGF/p75: Lens-epithelium-derived growth factor
PIC: Preintegration complex
PML: Promyelocytic leukemia protein
PTMs: Posttranslational modifications
SIM: SUMO-interacting motif
SUMO: Small ubiquitin-like modifier
WB: Western blot
